# Quantification of myocardial stiffness in heart failure with preserved ejection fraction porcine model using magnetic resonance elastography

**DOI:** 10.1186/1532-429X-18-S1-P29

**Published:** 2016-01-27

**Authors:** Ria Mazumder, Samuel Schroeder, Bradley D Clymer, Richard D White, Arunark Kolipaka

**Affiliations:** 1 Electrical and Computer Engineering, The Ohio State University, Columbus, OH USA; 2 Department of Radiology, The Ohio State University, Columbus, OH USA; 3 Department of Mechanical Engineering, The Ohio State University, Columbus, OH USA

## Background

Heart failure with preserved ejection fraction (HF*p*EF) is associated with a complex heterogeneous pathophysiology which is poorly understood thereby preventing appropriate diagnosis and prognosis^1^. However, it is known that most of the cardiovascular and non-cardiac abnormalities that induce HF*p*EF are eventually manifested as an increase in left ventricular (LV) myocardial stiffness (MS). Therefore, we hypothesize that quantifying MS will assist in timely diagnosis of HF*p*EF and reveal pathophysiological conditions that are specific to the prognosis of HF*p*EF. Recently, with the advent of cardiac magnetic resonance elastography (cMRE) it has been feasible to estimate the shear stiffness of myocardium noninvasively^2^. In this study, we use cMRE to estimate the change in LV MS across the cardiac cycle during disease progression in HF*p*EF induced pigs.

## Methods

Renal wrapping surgery was performed in 5 pigs to induce HF*p*EF^3^. cMRE was performed at baseline (Bx, prior to surgery), month 1 (M1) and month 2 (M2) on a 1.5T MRI scanner (Avanto, Siemens Healthcare, Erlangen, Germany). cMRE imaging parameters includes: TR/TE = 12.5/9.71 ms; FOV = 384 × 384 mm^2^; Resolution = 3 × 3 × 8 mm^3^; Flip angle = 15°; GRAPPA = 2; Mechanical frequency = 80 Hz; Encoding frequency = 160 Hz; Phase offsets = 4. Images were masked to extract the LV and estimate cMRE-derived LV MS across the cardiac cycle using 3D local frequency estimation inversion algorithm at each time point. End- systolic (ES) and diastolic (ED) MS were correlated to the corresponding ES and ED pressures obtained using LV catheterization.

## Results

**Figure 1:**
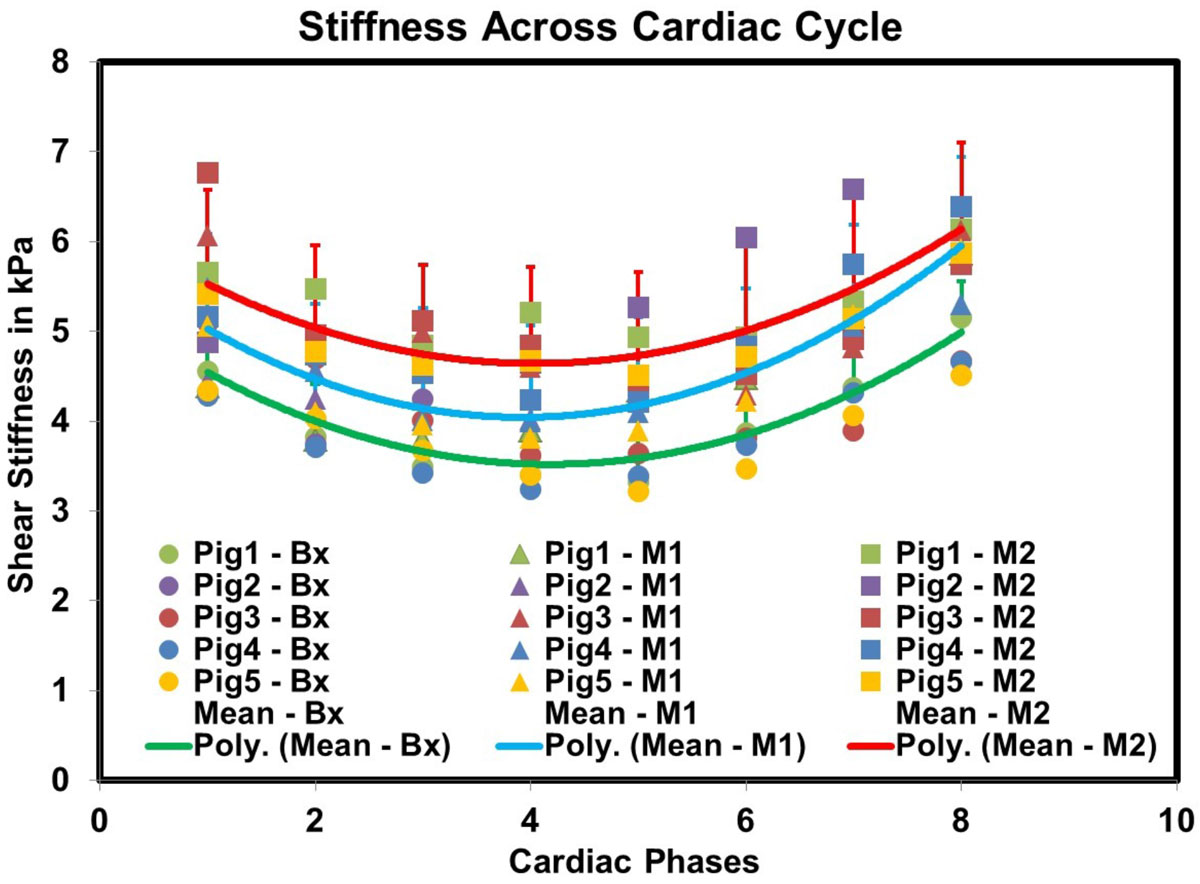
**Plot of cMRE-derived MS across the cardiac cycle from all animals showing that mean MS at M2 (red line) increased from M1 (blue line) which increased compared to Bx (green line)**. ED occurred between 4 and 5 while ES occurred between 8 and 1.

**Figure 2:**
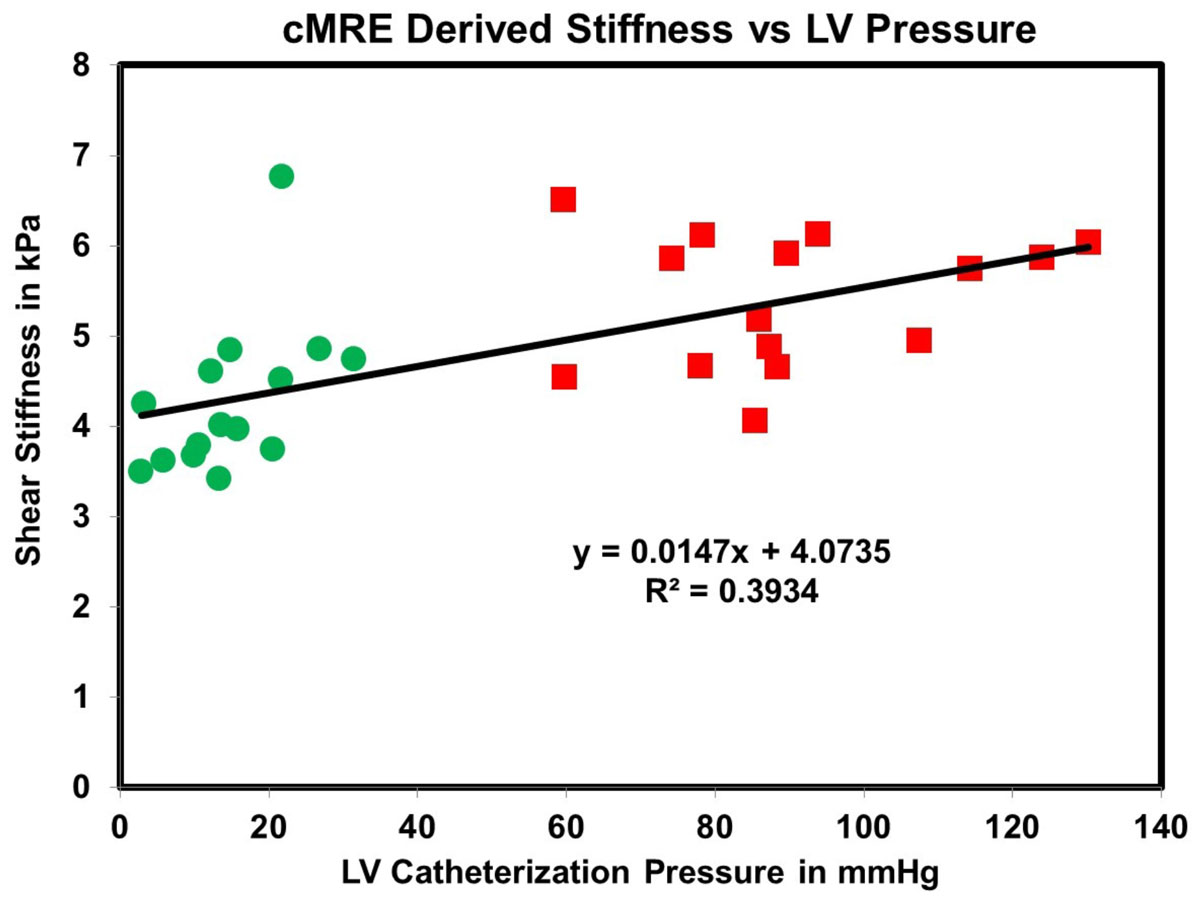
**Graph shows correlation between cMRE-derived ES and ED MS vs catheter-based ES and ED LV pressure**.

Fig 1 demonstrates that cMRE-derived stiffness increases with disease progression from Bx to M1 to M2 throughout the cardiac cycle indicating that HF*p*EF causes an elevation in LV MS. Furthermore, ES MS is significantly higher (p-value < 0.001) than ED MS at all-time points. Fig 2 shows moderate correlation between ES and ED MS and corresponding pressure from LV catheterization with a R^2^ value of 0.4.

## Conclusions

Our result demonstrates that cMRE-derived MS increases with disease progression in HF*p*EF porcine model thereby indicating the scope of using cMRE as a diagnostic tool to diagnose HF*p*EF condition.
